# Chemical probes and inhibitors of bromodomains outside the BET family†
†The authors declare no competing interests.


**DOI:** 10.1039/c6md00373g

**Published:** 2016-09-07

**Authors:** Moses Moustakim, Peter G. K. Clark, Duncan A. Hay, Darren J. Dixon, Paul E. Brennan

**Affiliations:** a Department of Chemistry, University of Oxford, Oxford OX1 3TA, UK; b Structural Genomics Consortium, University of Oxford, OX3 7DQ, UK. Email: paul.brennan@sgc.ox.ac.uk; c Target Discovery Institute, Nuffield Department of Medicine, University of Oxford, OX3 7FZ, UK; d Department of Chemistry, Simon Fraser University, Burnaby V5A 1S6, Canada; e Evotec (UK) Ltd, 114 Innovation Drive, Milton Park, Abingdon, Oxfordshire OX14 4RZ, UK

## Abstract

In the last five years, the development of inhibitors of bromodomains has emerged as an area of intensive worldwide research. Emerging evidence has implicated a number of non-BET bromodomains in the onset and progression of diseases such as cancer, HIV infection and inflammation. The development and use of small molecule chemical probes has been fundamental to pre-clinical evaluation of bromodomains as targets. Recent efforts are described highlighting the development of potent, selective and cell active non-BET bromodomain inhibitors and their therapeutic potential. Over half of typical bromodomains now have reported ligands, but those with atypical binding site residues remain resistant to chemical probe discovery efforts.

## Abstract 2

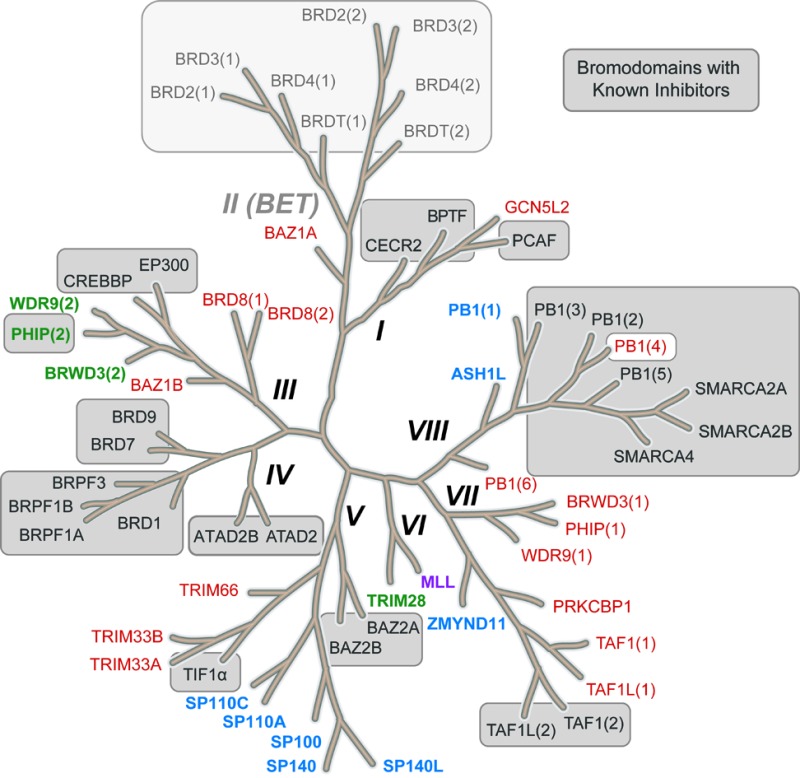
Significant progress has been made in discovering inhibitors and chemical probes of bromodomains, epigenetic readers of lysine acetylation.

## Introduction

**Fig. 1:**
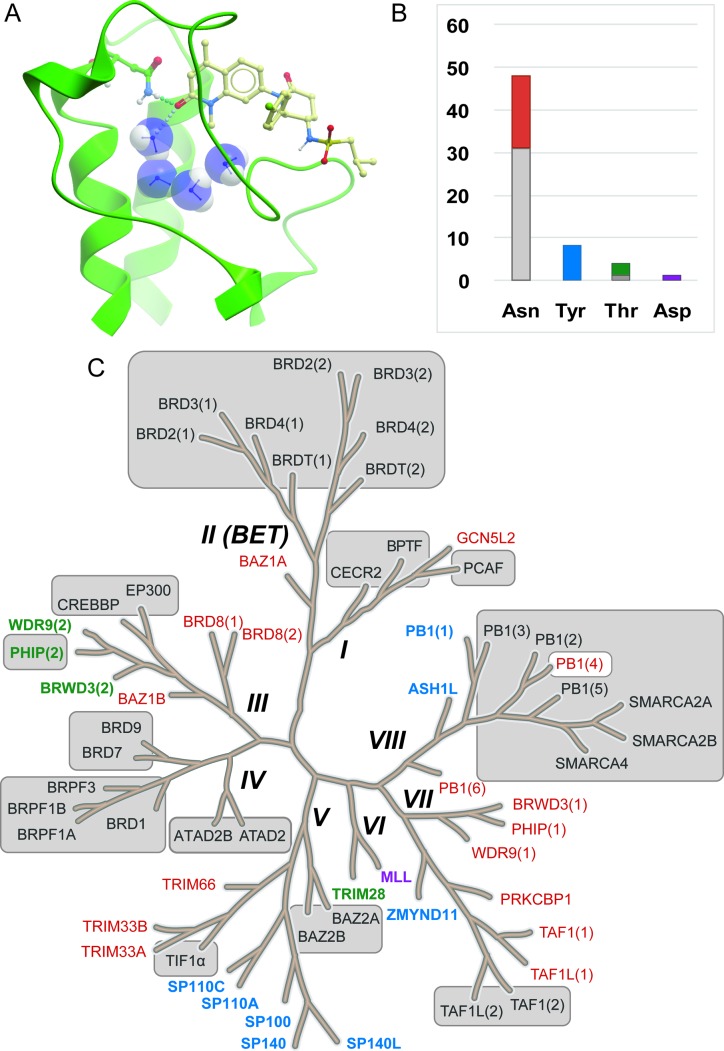
Bromodomains with reported inhibitors. A. Brd inhibitors such as LP99 (pale ball and stick) bind in the acetyl lysine binding pocket (green ribbon) to a common Asn residue (green ball and stick) and a network of water molecules (blue CPK) (LP99 and BRD9 from PDB ID 5IGN). B. Distribution of acetyl lysine binding residues in Brd pockets. Brds are colored by their acetyl lysine binding residue (red: Asn, blue: Tyr, green: Thr, purple: Asp). All reported Brd inhibitors (grey sections of bars) target Brds with a typical Asn residue with the exception of PHIP(2) which has a Thr. C. Brds in grey boxes have reported inhibitors. Brds in colored typeface have no reported inhibitors (colors as in B).

Epigenetics describes the network of mechanisms that modulates gene expression without directly affecting gene sequence.^1^ A number of proteins are involved in epigenetic control and referred to as readers (bromodomains, chromodomains, tudor domains, *etc.*), writers (lysine acetyltransferases, lysine methyltransferases and DNA methyltransferases) and erasers (lysine deacetylases and lysine demethylases). These proteins interact with and act upon DNA or histones (large nuclear proteins which DNA is packaged around), and non-histone proteins such as transcription factors. They function by adding, removing and interacting with epigenetic marks (post-translational modifications to histone proteins). Aberrant regulation of a number of these epigenetic proteins is linked with the onset and progression of multiple disease states including cancer^2–4^ and inflammation.^5^ Lysine acetylation, which is effected by the lysine acetyltransferases (KATs) and removed by lysine deacetylases (KDACs), is an epigenetic mark that has been the subject of a plethora of research.^6–9^ Bromodomains (Brds) bind to acetylated lysines (KAc) in histones and other proteins through the bromodomain KAc binding site which is also the binding site of most Brd ligands (Fig. 1A).

Once bound to acetylated histones, bromodomains recruit other nuclear proteins to form large chromatin modelling and transcriptional regulation complexes. To this end, bromodomains are increasingly being considered as attractive therapeutic targets for a variety of disease states due to the critical role they play in control of target genes that are difficult to modulate directly with small molecules.^10^

The 61 Brds in the human genome can be divided into eight subfamilies based on their sequence and structures (Fig. 1C).^8^ Brds can be further classified by a single key residue in the binding site. The majority of Brds have an Asn as the KAc recognition residue (48 examples), but a minority have either a Tyr (8), Thr (4) or Asp (1) residue (Fig. 1B). To date, all potent inhibitors target one of the typical Asn-containing Brds (Fig. 1B and C).

To date a large majority of the work in validating the pharmacological relevance of Brds as therapeutic targets has relied upon genetic manipulation of an entire Brd containing gene *via* knock-out or RNAi knock down. It should be noted that this does not imply modulation of individual Brds will deliver a pharmacological effect or phenotype. However, the development of chemical probes and inhibitors for Brds will afford the scientific community with an additional ‘go/no-go’ checkpoint on implicated Brds in target validation. Use of chemical probes from multiple chemotypes will also be of benefit as this will allow for a more robust analysis of Brd inhibition and pharmacological effect due to the likely orthogonal off-target activity of different chemical series.

A chemical probe has been defined to be an entity capable of binding to a given target with *in vitro* potency <100 nM (*K*_*D*_ or IC_50_), selectivity >30-fold against other families and evidence of cellular target engagement <1 μM compound concentration.^11^ As research in this area has progressed, additional desirable features are becoming necessary for qualification of a small molecule entity as a chemical probe such as availability of a negative control compound, favourable toxicity profiles, higher selectivity (*e.g.* intra-family and >100 fold over BET Brds – for non-BET bromodomain chemical probes).^12^ It is expected that as the field develops, the delivery of chemical tools that satisfy these criteria to a greater extent may drive more demanding criteria for what is deemed a chemical probe. Brds that have few to no chemical probes may be associated with slightly relaxed chemical probe criteria, to allow for rapid dissemination of early chemical probe material and associated data (thereby promoting developments in the understudied target). Where compounds have fallen short of satisfying one or more key chemical probe criterion – owing to deficiencies in the chemical entity or missing data, they are termed ‘inhibitors’ (the application of this term may be applied more regularly for target areas where higher quality chemical probes already exist). Collectively in this review both chemical probes and inhibitors are referred to as ‘chemical tools’.

Early studies into the development of chemical tools useful in interrogating bromodomain function yielded many probes and inhibitors of the BET bromodomains (sub-family II: BRD2, BRD3, BRD4, BRDT) (Fig. 1C). Previously written reviews have discussed the discovery and impact of such chemical tools in some depth, in part owing to the significant pharmacological relevance of targeting the BET bromodomains.^7,13–18^ This review will focus on chemical probes^11,12^ and inhibitors of the remaining non-BET bromodomains^14,17,18^ of the Brd sub-families I and III–VIII. Where possible selectivity over BET Brds will be discussed which is deemed a critical factor in interpreting the effects of Brd inhibitors, this can be rapidly ascertained through a number of tractable assays including Differential Scanning Fluorimetry (DSF) selectivity panels.^19^ An additional desirable feature of chemical probes/inhibitors would be achieving different degrees of selectivity: family-wide inhibition and intra-family selectivity as comparisons of inhibition profiles would allow for an accurate analysis of Brd pharmacological relevance (family-wide relevance *vs.* specific Brd relevance). Other properties of chemical probes and inhibitors are discussed herein if known, such as cellular activity, pharmacokinetics and solubility.

## Sub-family I

### PCAF

A number of reports have provided support for the therapeutic potential in the development of inhibitors of the PCAF Brd (p300/CBP Associated Factor) owing to the link with a variety of diseases including cancer,^20–22^ HIV,^20,23–26^ and neuro-inflammation^20,27^ PCAF has been predicted to be a highly druggable target by Vidler *et al.* ^28^

**Fig. 2:**
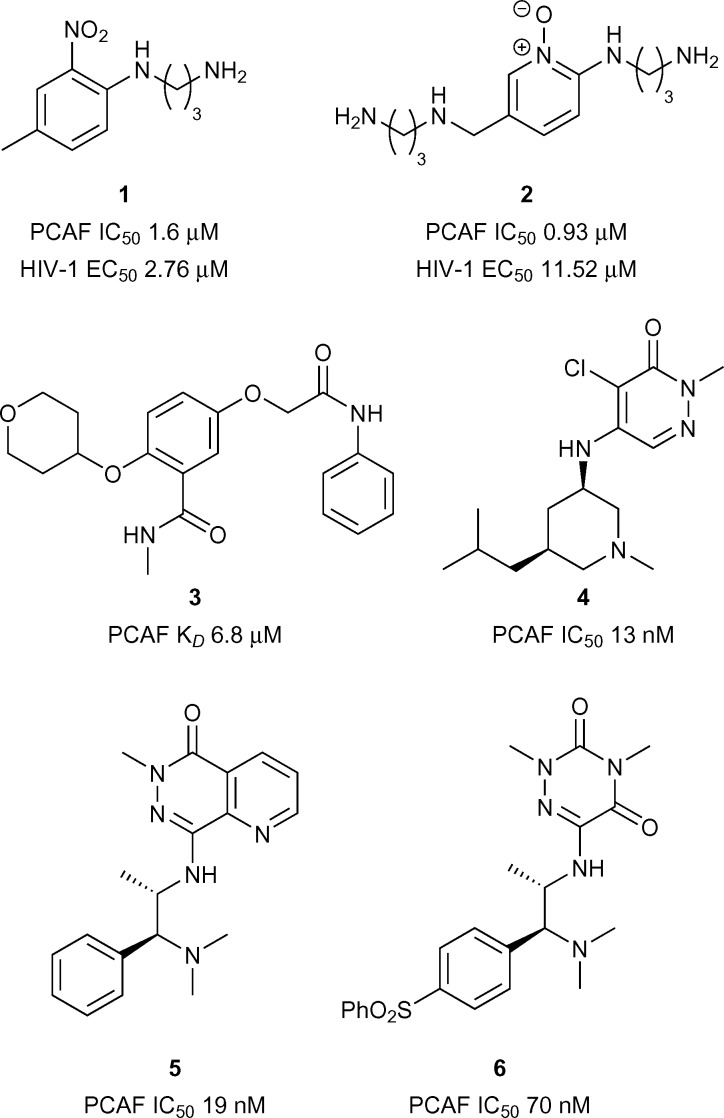
PCAF bromodomain inhibitors.

Early reports of PCAF bromodomain inhibitors were disclosed by Wang *et al.* ^25^ It was shown that interactions between the HIV-1 Tat peptide (a viral factor essential for replication) and PCAF Brd were disrupted through competitive binding to PCAF bromodomain by compound **1** (reference compound **16**) (PCAF IC_50_ 1.60 μM, Fig. 2). Compound **1** was also shown to be effective at perturbing HIV-1 replication (EC_50_ 2.76 μM). Hu *et al.* ^29^ later reported on more PCAF bromodomain inhibitors including compound **2** (reference compound **20**) which displayed moderate to good inhibition against PCAF Brd/Tat association (IC_50_ 0.93 μM, Fig. 2) and viral replication (EC_50_ 11.52 μM). Recently Chaikuad *et al.* at the Structural Genomics Consortium (SGC) discovered fragment leads for the PCAF Brd. Compound **3** (reference compound **14**) showed moderate binding activity using Isothermal Titration Calorimetry (ITC) (PCAF *K*_*D*_ 6.80 μM, Fig. 2).^30^ More recently Genentech and Constellation pharmaceuticals disclosed the structures of highly potent compounds **4**, **5** and **6** (reference examples 20, 65B and 18 respectively) (IC_50_ 19 nM and 70 nM respectively, Fig. 2) for the treatment of PCAF mediated diseases including cancer.^21,22,31^

### CECR2

**Fig. 3:**
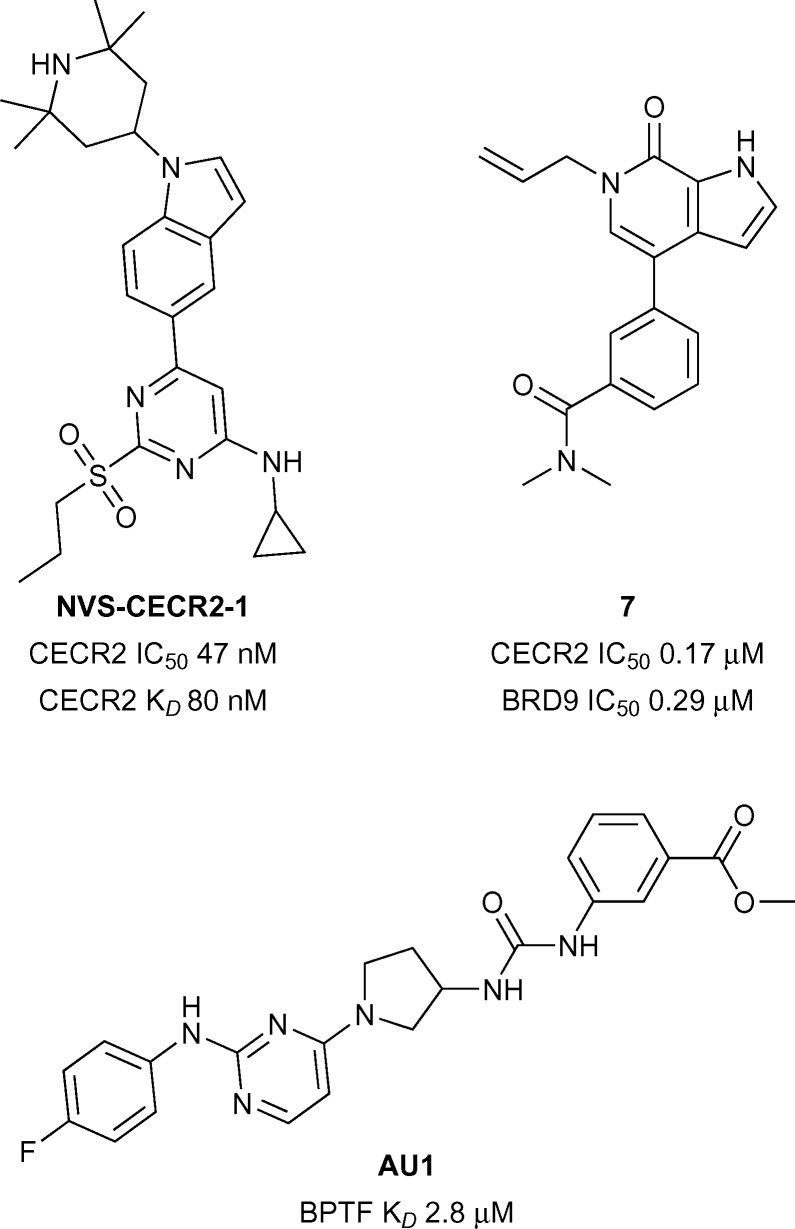
CECR2/BPTF inhibitors.

CECR2 has been predicted to be a highly druggable target.^28^ A highly potent and selective chemical probe for the bromodomain containing CECR2 (Cat Eye Syndrome Chromosome Region, candidate 2) has recently been developed by Novartis and the SGC (**NVS-CECR2-1**, Fig. 3).^32^ Details of the development of this probe is yet to be published, however **NVS-CECR2-1** is reported to have high affinity for CECR2 (CECR2 IC_50_ 47 nM, CECR2 *K*_*D*_ 80 nM). **NVS-CECR2-1** also displays robust in-cell target engagement in a Fluorescence Recovery After Photobleaching (FRAP) assay at 0.1 μM against full-length CECR2, despite being poorly soluble.^32^ Co-workers from Genentech and Constellation pharmaceuticals have recently reported the development of inhibitors of TAF1(2), CECR2, BRD4(1) and BRD9 from a common *N*-methyl pyrrolopyridone structural motif.^33^ Compound **7** (reference compound **3**, Fig. 3) was shown to inhibit the CECR2 bromodomain with good potency (CECR2 IC_50_ 0.17 μM) and exhibited novel interactions stemming from the rearrangement of the conserved solvent network. Compound **7** was profiled for selectivity across a broad range of bromodomain targets (DiscoveRx BROMO*scan*)^34^ showing significant off-target potency for BRD9 (BRD9 *K*_*D*_ < 0.1 μM) as confirmed by a Time-resolved Förster resonance energy transfer assay (TR-FRET) (BRD9 IC_50_ 0.29 μM).^35^

### BPTF

BPTF (Bromodomain and PHD Finger Transcription Factor/FALZ) has been linked to various cancers including bladder,^36^ colorectal,^37^ melanoma,^38^ and leukemia.^39^ BPTF has been predicted to be a highly druggable target.^28^ Until recently no known inhibitors of BPTF had been available to interrogate its role in the onset and progression of cancer. Urick *et al.* have recently reported the first BPTF inhibitor (**AU1**, Fig. 3) discovered through a ^19^F NMR assay.^40^ Moderate potency (BPTF *K*_*D*_ 2.8 μM) was displayed by **AU1** *in vitro* and in a cell-based reporter assay. Further optimisation of the chemical scaffold of **AU1** may serve as a good strategy towards more potent BPTF inhibitors.

## Sub-family III

### CBP/p300

The lysine acetyltransferases CBP (also known as CREBBP and KAT3A) and p300 (also known as EP300 and KAT3B) are among the most studied bromodomain-containing proteins outside of the BET sub-family.^41–45^ CBP and p300 share a high degree of sequence similarity, particularly in their bromodomains (96% similar).^46^ Interest in developing chemical probes for CBP/p300 has been fuelled by the large and diverse nature of cellular processes which utilise these transcriptional coactivators, and by the strong links of CBP and p300 dysfunction with human disorders and diseases. In particular, CBP and p300 are implicated in the developmental disorder Rubinstein–Taybi syndrome,^47^ and are strongly linked to cancer, especially haematological malignancies,^48^ inflammation,^49^ and neuropsychiatric disorders.^50^

**Fig. 4:**
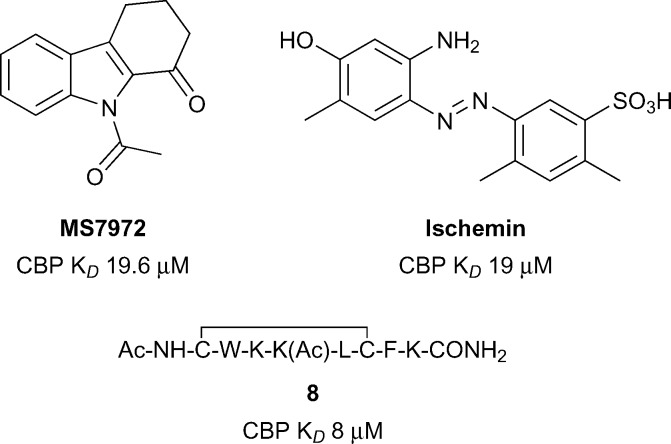
Early CBP inhibitors from the Zhou group.

The Zhou group pioneered the development of inhibitors of the CBP bromodomain (Fig. 4).^51^ The *N*-acetyl indole, **MS7972**, had modest CBP bromodomain affinity (CBP *K*_*D*_ 19.6 μM) and inhibited the association of acetylated p53 with the CBP bromodomain at 50 μM.^51–53^ A biarylazo inhibitor, **Ischemin**, also displayed modest CBP bromodomain affinity (CBP *K*_*D*_ 19 μM) and inhibited p53-induced p21 activation in a reporter-gene assay (p21 IC_50_ 5 μM).^52^ The cyclic peptide **8** (reference compound **4**) has also been shown to bind the CBP Brd (*K*_*D*_ 8 μM) and to inhibit p53 activation in a reporter assay.^52^ The inhibitors reported by the Zhou group demonstrated that the CBP bromodomain could be targeted by multiple diverse chemotypes. However, the utility of these early ligands was limited by their relatively low affinity and lack of reported selectivity data for other bromodomain subfamilies.

**Fig. 5:**
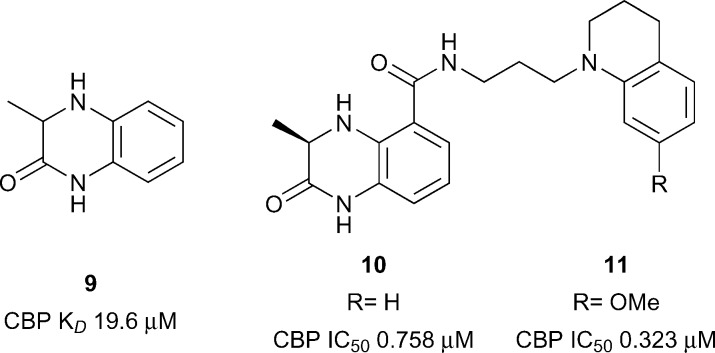
Dihydroquinoxalinone CBP inhibitors.

The first reported sub-micromolar CBP ligands were described by Rooney and co-workers.^54^ A series of dihydroquinoxalinones was developed from a fragment hit. The fragment itself was discovered after compound **9** was found to be a weak (CBP IC_50_ 1.9 mM, Fig. 5) but efficient ligand for CBP (ligand efficiency 0.54). Screening of *N*-methylpyrrolidinone (NMP) analogues led to two series being pursued: benzoxazinones and dihydroquinoxalinones. An optimised dihydroquinoxalinone inhibitor, compound **11** (reference compound (*R*)-2) was shown to have sub-micromolar affinity for CBP (*K*_*D*_ 0.39 μM), albeit with modest selectivity over BRD4(1) (*K*_*D*_ 1.4 μM). An X-ray structure of compound **10** (reference compound (*R*)-1) bound to CBP revealed that the dihydroquinoxalinone moiety mimics the acetyl lysine binding interactions, whilst an internal hydrogen bond helps to direct the tetrahydroquinoline moiety into an induced-fit pocket created by the movement of R1173 to allow a cation–π interaction arginine side chain. Molecular dynamics were used to calculate the contribution of the cation–π interaction (3.2–4.7 kcal mol^–1^). On target cellular activity was shown in a FRAP assay, where dose-dependent inhibition of the FRAP signal of a GFP-tagged CBP construct with SAHA-stimulated hyper-acetylated chromatin was observed for compound **11** at low micromolar concentrations.

**Fig. 6:**
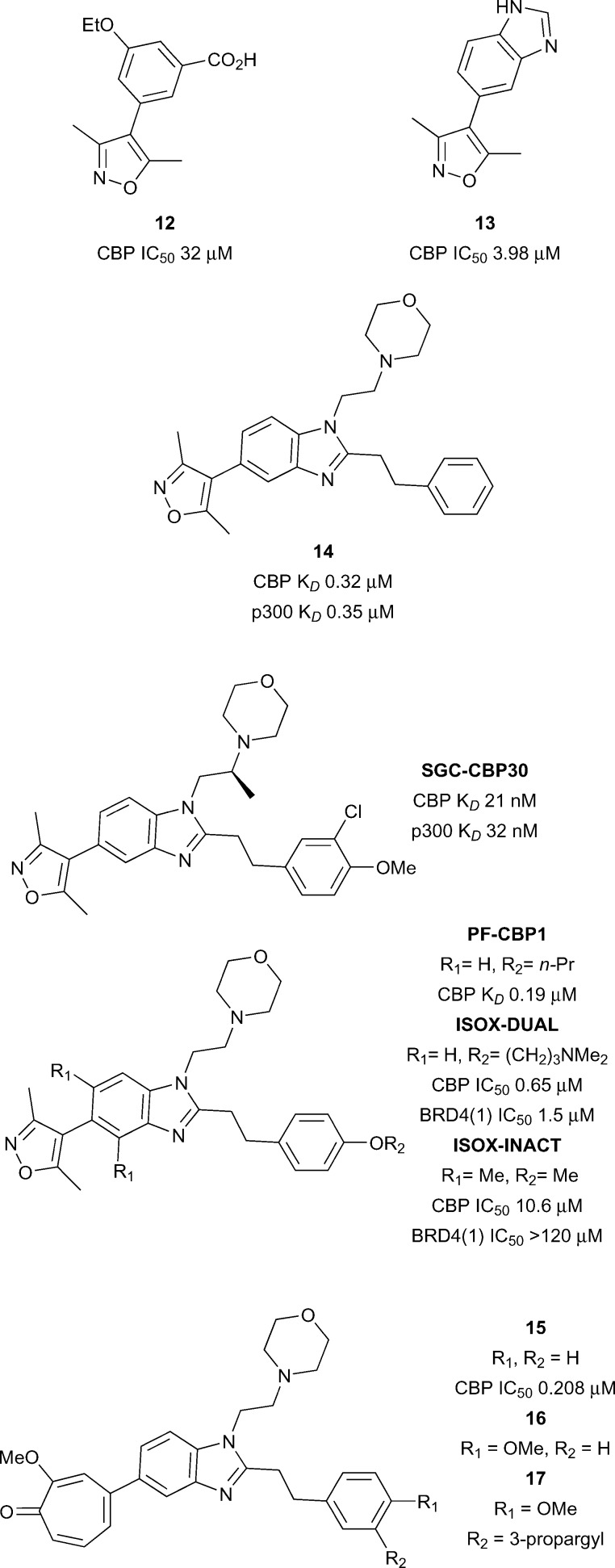
Isoxazole and related CBP/p300 inhibitors.

Hewings and co-workers gave further encouragement that selective CBP bromodomain inhibition was possible with small molecules (Fig. 6).^55^ A series of 4-aryl-3,5-dimethylisoxazoles was described with differing selectivity for the Brd sub-families. Compound **12** inhibited a histone peptide–Brd interaction at micromolar concentrations in an AlphaScreen assay (CBP IC_50_ 32.2 μM) and had modest selectivity (1.6-fold) for CBP over the first Brd of BRD4 (BRD4(1)). X-ray crystallography revealed that the dimethylisoxazole acts as the KAc mimic *via* a direct hydrogen bond between the isoxazole oxygen and the amide side-chain of N1168, and a water-mediated hydrogen-bond to Y1125 from the isoxazole nitrogen. The ethoxy oxygen of compound **12** forms a hydrogen bond to another structured water in the ZA channel. Possible weak electrostatic interactions between the carboxylate of **12** and R1173 may be partly responsible for the CBP selectivity. The discovery of more potent and selective CBP/p300 inhibitors soon followed.

The discovery of the CBP/p300 Brd chemical probe **SGC-CBP30** began with a 5-isoxazolyl-benzimidazole fragment **13** (Fig. 6) which was unselective for CBP over BRD4(1).^46^ *N*-1 and *C*-2 substituents were introduced to target regions of structural difference between CBP and BRD4(1). The combination of a phenethyl group at the C-2 position and an ethylene linked morpholine at *N*-1 gave compound **14** (reference compound **17**) displaying sub-micromolar affinity for CBP and p300 as measured by ITC (CBP *K*_*D*_ 0.32 μM and p300 *K*_*D*_ 0.35 μM). However **14** also displayed off-target activity against BRD4(1) (3-fold selectivity against CBP *vs.* BRD4(1)). Analysis of the X-ray structure of compound **14** bound to CBP and BRD4(1) guided the design of more potent and selective inhibitors. Potency was initially enhanced through variation of the substitution on the phenyl ring. Attempts at rigidifying the scaffold to increase selectivity led to the observation that introduction of a methyl branch on the *N*-1 ethylene linker led to potent and selective analogues. When synthesised as single enantiomers, it was found that the (*S*)-methyl analogues were favourable for CBP binding. The optimal compound (**SGC-CBP30**) was found to have low nanomolar affinity for CBP and p300 (CBP *K*_*D*_ 21 nM and p300 *K*_*D*_ 32 nM) and also displayed 40-fold selectivity for CBP over BRD4(1). **SGC-CBP30** was also shown to be selective against a wide panel of bromodomain subfamilies in a Differential Scanning Fluorimetry (DSF) assay.^19^ X-ray crystallography of SGC-CBP30 bound to CBP revealed the expected dimethylisoxazole interactions with the acetyl lysine binding residues, whilst the aryl group formed a cation–π interaction with R1173 in an induced pocket analogous to that observed by Rooney *et al.* ^54^ On-target cellular activity was demonstrated in a FRAP assay and a p53 reporter assay (p53 IC_50_ 1.54 μM). More recently, **SGC-CBP30** has been shown to reduce immune cell production of pro-inflammatory cytokines, including IL-17A, and to inhibit IL-17A secretion from Th17 cells.^56^ Transcriptional profiling of **SGC-CBP30** in T cells indicated that the effects of CBP/p300 bromodomain inhibition were more limited than those of pan-BET inhibitor JQ1. The report suggests that inhibition of CBP and p300 bromodomains warrants further investigation as a potential therapeutic strategy to combat ankylosing spondylitis, psoriatic arthritis and other human type-17-mediated autoimmune diseases.

A team at Pfizer modified the **SGC-CBP30** scaffold with the aim of improving the selectivity for CBP over BRD4(1).^57^ A propoxy analogue of **SGC-CBP30**, **PF-CBP1** (Fig. 6), was found to have good affinity for CBP (CBP *K*_*D*_ 0.19 μM) and was selective over BRD4(1) (BRD4(1) *K*_*D*_ 20 μM). BROMO*scan* (DiscoveRx) profiling confirmed the broader selectivity of **PF-CBP1** for CBP and p300 over other bromodomain subfamilies. The report also describes a dual CBP/BRD4(1) inhibitor, **ISOX-DUAL** (CBP IC_50_ 0.65 μM BRD4(1) IC_50_ 1.5 μM). A negative control was developed by introduction of two methyl groups, flanking the dimethylisoxazole head group, which cause an unfavourable change in the isoxazole-benzimidazole torsion angle. The resulting compound, **ISOX-INACT**, was a very weak inhibitor of CBP and was inactive against BRD4(1) (CBP IC_50_ 10.6 μM, BRD4(1) IC_50_ > 120 μM). Computational techniques have been used to search for bioisosteric replacements for the dimethylisoxazole head group on the **SGC-CBP30** scaffold.^58^ It was found that tropolone benzimidazole, compound **15** (reference compound **3**) was a sub-micromolar inhibitor of CBP and BRD4(1) (CBP IC_50_ 0.208 μM, BRD4(1) IC_50_ 0.343 μM). An X-ray crystal structure of **15** bound to CBP and BRD4(1) confirmed that the tropolone head group was acting as the acetyl lysine mimic. The tropolone head group was seen as an attractive photoreactive handle for capture of bromodomains. Treatment of CBP and BRD4(1) with the *para*-methoxy derivative **16** (reference compound **4**) with irradiation at 365 nm led to the observation of a MS adduct of protein plus inhibitor, minus 14 Da, presumably formed by photoreaction of the ligand with the protein and demethylation. The alkyne derivative **17** was prepared (reference compounds **7**) in order to give a ‘clickable’ handle and was used to photolabel BRD4(1) spiked into K562 cells. Subsequent copper-mediated azide-alkyne cycloaddition to an azide-biotin tag and streptavidin-enrichment led to visualisation of the labelled BRD4(1) by Western blot. Although no native BRD4 could be isolated from live cells using the same method, the approach shows the potential for modification of chemical probes to provide photoreactive tools for the capture of bromodomain-containing proteins. A complementary CBP/p300 bromodomain chemical probe from an alternative chemotype has been disclosed by the SGC and GSK.

**Fig. 7:**
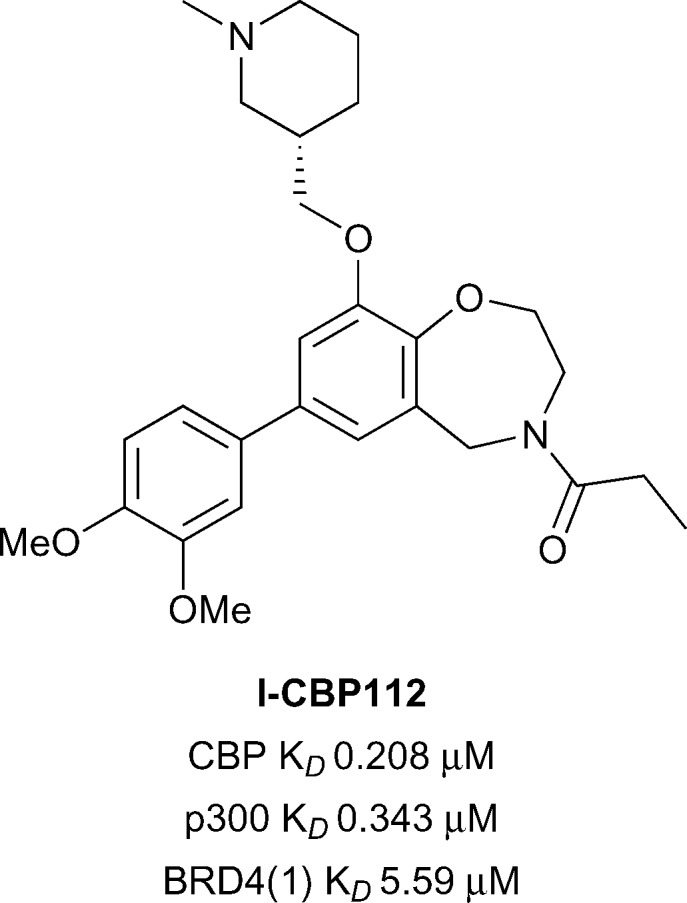
CBP/p300 chemical probe **I-CBP112**.

Oxazepine **I-CBP112** (Fig. 7), was discovered through the analysis of inhibitors which were structurally related to the BET-selective inhibitors JQ1 and I-BET762.^59^ **I-CBP112** was found to be highly selective in a DSF selectivity panel of 41 other bromodomains, and in a biolayer interferometry (BLI) panel of 42 bromodomains. Weak off-target activity was noted only for the BET sub-family of bromodomains. The affinity of **I-CBP112** for CBP and p300 was measured by ITC (CBP *K*_*D*_ 0.151 μM, p300 *K*_*D*_ 0.167 μM). **I-CBP112** displayed 37-fold selectivity for CBP over BRD4(1) (BRD4(1) *K*_*D*_ 5.59 μM). An X-ray crystal structure of the (*R*)-enantiomer of **I-CBP112** bound to CBP confirmed that the carbonyl group mimicked the KAc binding interactions. The aromatic group formed a π–π interaction with R1173 in an induced pocket also seen for **SGC-CBP30** and **14**. On target cellular activity was demonstrated in a FRAP assay, where **I-CBP112** significantly reduced the recovery time using a GFP-tagged triple-CBP bromodomain substrate. In a nanoBRET assay **I-CBP112** inhibited the interaction of a nanoLuc luciferase CBP bromodomain construct with a Halo-tagged histone H3.3 construct (CBP IC_50_ 0.6 μM).^60^ **I-CBP112** was also screened for novel phenotypes in the DiscoveRx BioMAP Diversity PLUS panel where altered expression of the anti-inflammatory cytokine IL10 and VCAM1 was observed.^61^ **I-CBP112** was found to reduce the clonogenic growth of MLL-CBP immortalised murine bone marrow cells, but did not significantly affect the cell survival. Treatment of MLL-AF9^+^ leukemic myeloblasts with **I-CBP112** reduced the number of leukemic stem cells. Additionally, transplantation of MLL-AF9^+^ cells pre-treated with **I-CBP112** delayed the disease initiation. Furthermore **I-CBP112** was shown to impair the clonogenic growth of 12 cells from 12 human leukemic cell lines and to reduce the number of colonies in primary human AML cells. In combination with BET inhibitor JQ1, **I-CBP112** was also found to enhance the cytotoxic effects of doxorubicin in human leukemic cells.

The availability of multiple chemotypes with the ability to inhibit a particular target affords greater confidence in the interpretation of results from cellular and *in vivo* studies because the chances of spurious off-target effects are reduced. Reports have begun to emerge utilising **SGC-CBP30** and **I-CBP112** in concert to interrogate CBP/p300 biology. Researchers at Genentech demonstrated that **SGC-CBP30** and **I-CBP112** caused a slowdown in the proliferation of multiple myeloma cells through arrest in the G1 cell cycle phase.^10^ RNA sequencing of **SGC-CBP30** treated LP-1 cells indicated that IRF4 (interferon regulatory factor 4) target genes were down-regulated. Notably, the gene encoding for Myc was down-regulated which has been shown to be important for cell division and growth. IRF4 is essential for the survival of multiple myeloma cells, indicating that the IRF4/Myc transcription pathway is being affected by CBP/p300 bromodomain inhibition. The results suggest that CBP/p300 bromodomain inhibition has therapeutic potential for the treatment of multiple myeloma. A Genentech patent also investigated the effects of **SGC-CBP30** and **I-CBP112** BET inhibitor resistant cells.^62^ The BET inhibitor resistant cells were generated by treating acute myeloid leukemia cells (NOMO-1) with increasing concentrations of a BET inhibitor. Treatment of the BET inhibitor resistant cells with **SGC-CBP30** or **I-CBP112** inhibited MYC expression and cell growth. The claims in the patent include independent and synergistic dosing of CBP/p300 and BET inhibitors to treat a number of human malignancies.

**Fig. 8:**
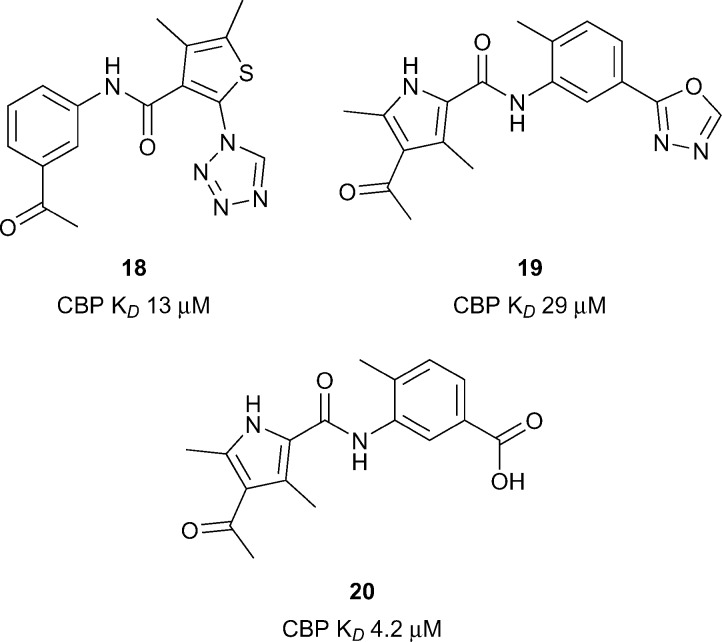
CBP inhibitors from Nevado and Caflisch.

**Fig. 9:**
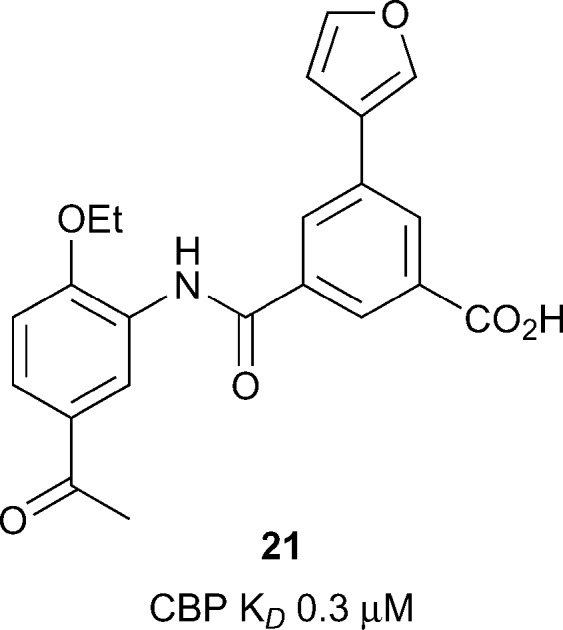
Optimized CBP inhibitor from Nevado and Caflisch.

Xu *et al.* utilised a computational technique coined ALTA (anchor-based library tailoring) to virtually screen a library of fragments against the CBP Brd.^63^ Two X-ray structures of CBP bearing different orientations of the sidechains of V1174 (the gatekeeper) and R1173 were used for the virtual screen. Molecules which contained the top ranking fragments were then docked. After clustering based on the ‘head groups’ which interacted with the key acetyl lysine-binding residues, **20** compounds were selected for molecular dynamics (MD) simulations. The simulations eliminated **3** out of the **20** compounds which moved out of the binding site in <100 ns. The remaining 17 compounds were assessed by BROMOscan. Two compounds, **18** and **19** (Fig. 8, reference compounds **1** and **9**), containing an acylaryl head group had low micromolar affinities (CBP *K*_*D*_ 13 μM, 17 CBP *K*_*D*_ 29 μM respectively). Further MD simulations with compound **18** suggested that replacement of the oxadiazole ring with a negatively charged group may enhance the CBP affinity through the gain of electrostatic interactions with the guanidinium sidechain of R1173. By analogy a benzoic acid derivative, compound **20**, (reference compound **6**) displayed improved CBP potency (CBP *K*_*D*_ 4.2 μM). Due to its synthetic versatility, compound **18** was selected for further optimization.^64^ Screening of commercially available analogues of the initial docking hit identified an inhibitor containing a fumaric acid derived amide. Replacing the fumaric acid moiety with an isophthalic group led to sub-micromolar potency in a competition assay. X-ray crystallography confirmed the acidic group forms favourable polar interactions with R1173 on CBP. Interaction with the Brd was further enhanced through substitution on the benzene ring *ortho* to the acid moiety. The elaborated inhibitors showed improved potency epitomised by the furan-containing inhibitor **21** (Fig. 9), (reference compound **19**), which had sub-micromolar affinity as measured by ITC (CBP *K*_*D*_ 0.3 μM). The optimised inhibitors were selective for CBP and p300 in a panel of 7 bromodomains and, along with their methyl ester derivatives, demonstrated growth inhibition in MOLM-13, ML2 and HL-60 leukemia lines.

**Fig. 10:**
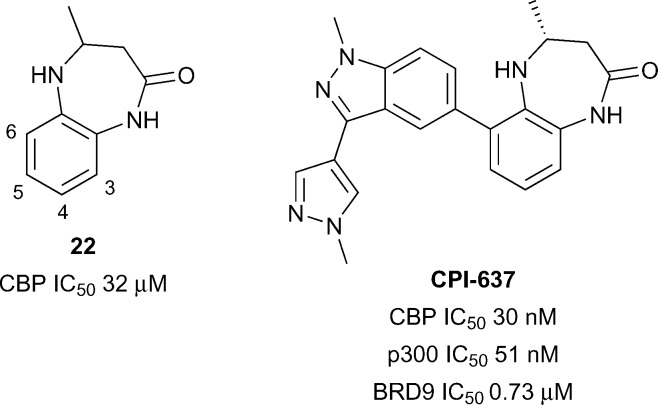
CBP/p300 inhibitors from Constellation and Genentech.

In another application of a fragment-based approach, Taylor *et al.* developed a benzodiazepinone fragment into a potent and selective inhibitor.^65^ The initial hit **22** (reference compound **1**, Fig. 10) was active against CBP (IC_50_ 32 μM) in a TR-FRET assay. Compound **22** was optimised while attempting to maintain a lipophilic ligand efficiency (LLE) consistent with moderate *in vivo* clearance. Substitution at the 6-position was used to orientate substituents along the LPF shelf (residues L1109-P1110-F1111), an approach which was hoped may lead to beneficial interactions with CBP and detrimental steric clashes with the corresponding WPF shelf in BRD4(1). In particular, it was found that 6-aryl substituents were beneficial for CBP potency and selectivity over BRD4(1). The optimised inhibitor **CPI-637** comprised a substituted indazole in the 6-position. The IC_50_ of **CPI-637** *versus* CBP and p300 was 30 nM and 51 nM respectively, while the selectivity over BRD4(1) was 367-fold and 215-fold. However off-target potency was observed against BRD9 (IC_50_ 0.73 μM). Target-related cellular activity was demonstrated in a CBP nanoBRET assay (EC_50_ 0.3 μM) and in the inhibition of MYC expression in AMO-1 cells (EC_50_ 0.60 μM).

Progress has clearly been made in the development of selective CBP/300 bromodomain inhibitors and there are are numerous chemotypes emerging as tool compounds. A common challenge that persists in the development of CBP/p300 inhibitors is selectivity over the BET subfamily. Non-selective compounds or poorly characterised compounds could confound interpretation of cellular studies as the effects of inhibiting the BET phenotype could be misattributed to CBP/p300 inhibition. The development of compounds with higher selectivity will help the field progress further. Nevertheless, careful cellular studies with SGC-CBP30 have revealed a distinct CBP/p300 phenotype.^10,46,56^

### PHIP

**Fig. 11:**
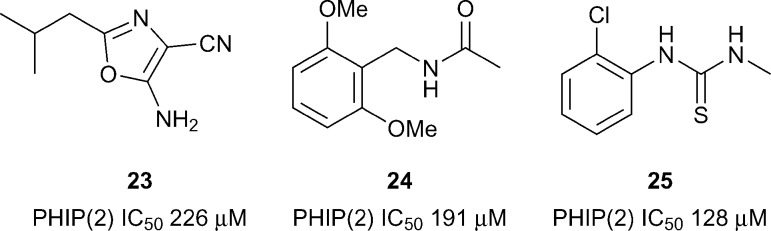
PHIP(2) fragment hits.

The only atypical Brd with a reported inhibitor is the second Brd of PHIP (PHIP(2)) which has a Thr instead of the typical Asn as the KAc recognition residue (Fig. 1B and C). PHIP is the most upregulated protein in metastatic melanoma and has potential as a therapeutic target and diagnostic marker.^66^ PHIP(2) has been predicted to be highly druggable.^28^ A high concentration crystallographic fragment was used to identify compounds **23–25** (reference compounds **4**, **12** and **11**, Fig. 11).^67^ Although binding of fragments **23–25** is very weak, they show that it is possible to find hits for the atypical Brds.

## Sub-family IV

### BRD7/9

Elucidation of the biological roles of BRD7 and BRD9 is an emerging area of research. Both BRD7 and BRD9 have been identified as members of the SWI/SNF nucleosome remodeling complex,^68,69^ which has emerged as an attractive target for developing anti-cancer agents.^70^ BRD7 is frequently found as a tumour suppressor,^71–73^ whereas BRD9 has been found to be mutated,^74^ upregulated,^75^ or over-expressed^76^ in various cancers. Additionally, BRD9 has been recently reported as a biomarker for Sézary syndrome,^77^ and for prediction of patient response to various forms of chemotherapy.^78^ Although the overall sequences of BRD7 and BRD9 share low similarity (36%), the homology in their Brds is significant (72%), a potential obstacle to the development of specific ligands. BRD9 has an intermediate druggability score according to analysis by Vidler *et al.*, however BRD7 was not assessed.^28,34^ The development of early BRD9 inhibitors has been recently reviewed,^79,80^ however further application of these probes in interrogating the activity of BRD7 and BRD9, in addition to the development of novel probes, has been reported subsequently.

**Fig. 12:**
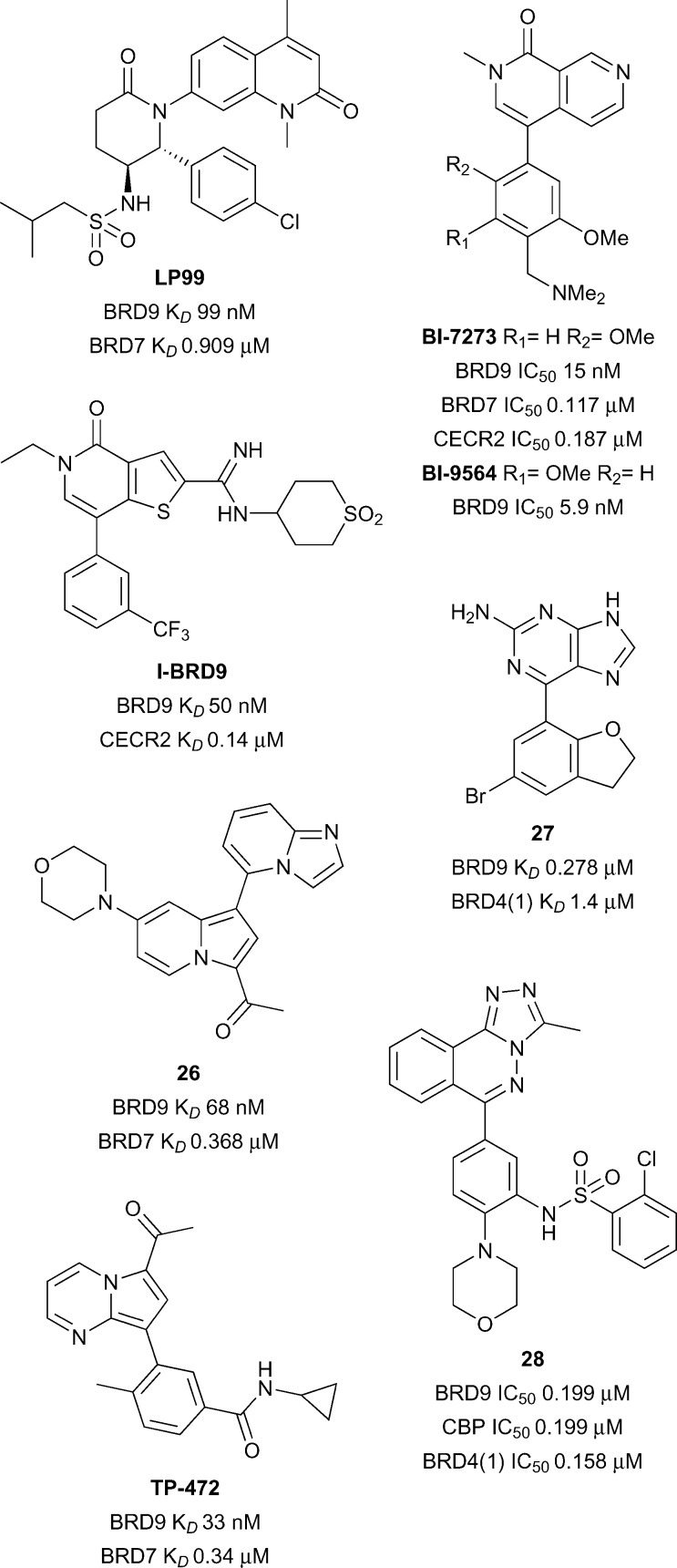
BRD7 and BRD9 inhibitors.

An initial dual BRD7/9 probe was developed by Clark and co-workers at the University of Oxford and the SGC. Fragment screening identified a quinolone lead that was subjected to a structure-based drug discovery program, culminating in the discovery of **LP99** (Fig. 12).^81^ This probe showed a high affinity for BRD9 (*K*_*D*_ 99 nM) with moderate activity against BRD7 (*K*_*D*_ 0.91 μM). Interactions with BRD9 were shown to be enthalpically driven with a net loss in entropy upon binding (Δ*H* –11 kcal mol^–1^, *T*Δ*S* –2.0 kcal mol^–1^), a finding supported by the determination of multiple H-bonding interactions in a co-crystal structure (Fig. 1A, PDB ID ; 5IGN): the carbonyl of the quinolone replicates the key H-bonds to N216 and Y173 of KAc recognition, with additional H-bonding *via* both the lactam carbonyl and the sulfonamide NH. One benefit of the chirality of **LP99** was the ready amenability of the opposite enantiomer as a negative control, as confirmed by ITC with no detectable inhibition of BRD9 observed. The selectivity of **LP99** was determined by DSF against all expressible Brds, in which, besides BRD9 and BRD7, no thermal shift >1.0 °C was observed. Cellular permeability and chromatin binding activity was confirmed using a FRAP assay, with U2OS cells expressing a full length BRD9-GFP fusion protein showing a dose-dependent decrease in fluorescence recovery times after **LP99** treatment. Cellular activity was further profiled through a nanoBRET assay with HEK293 cells expressing combinations of BRD7- or BRD9-NanoLuc fusion proteins and H3.3- or H4-HaloTag proteins: **LP99** decreased the BRET ratio for all combinations of BRD7/9 and H3.3/H4 in a dose-dependent manner (BRD7/9 IC_50_ 3.3–6.2 μM). Finally, a cytotoxicity assay performed with U2OS cells showed no effect of **LP99** on proliferation at concentrations below 33 μM.

Preliminary screening with **LP99** identified a role of BRD7/9 in inflammatory pathways. Initially, **LP99** was assessed for *in vitro* anti-cancer activity in the US National Cancer Institute human tumour cell line anticancer drug screen,^82^ although no growth inhibition of greater than 40% was observed for any cell line at a 10 μM concentration. Screening in a BioMAP panel, however, showed an effect of **LP99** on the secretion of pro-inflammatory cytokines. Interleukin 6 (IL-6) secretion from lipopolysaccharide-stimulated THP-1 monocytes was measured through an ELISA assay, with **LP99** resulting in a dose-dependent decrease in IL-6 secretion (IL-6 IC_50_ < 10 μM). This role of BRD7/9 in inflammatory pathways has been supported by a recent patent describing inhibition of BRD7 or BRD9 as a method for treating T_H_2 cytokine-mediated diseases.^83^

A BRD9-specific probe was concurrently developed by Theodoulou and co-workers at GlaxoSmithKline and the University of Strathclyde. An initial library screening revealed a thienopyridone lead against BRD9, which, through a structure-based drug discovery program, resulted in the development of **I-BRD9** (Fig. 12).^84^ A high affinity for BRD9 was observed through both a TR-FRET assay (IC_50_ 50 nM) and a BROMO*scan* assay (IC_50_ 1.9 nM), with the latter also demonstrating the selectivity of **I-BRD9**; >70-fold selectivity was seen against 34 Brds, including the homolog BRD7 (*K*_*D*_ 0.38 μM). Wider selectivity against 49 unrelated proteins, including ion channels, GPCRs, transporters, kinases, nuclear receptors and other enzymes, revealed IC_50_ values predominantly >10 μM, with limited activity seen on a serotonin receptor (IC_50_ 6 μM) and a norepinephrine transporter (IC_50_ 8 μM). An X-ray co-crystal structure of **I-BRD9** with BRD9 demonstrated the carbonyl of the thienopyridone replicating the key KAc interactions to N216 and Y173, with additional H-bonding to the protein by the amidine and a sulfone oxygen (PDB ID ; 4UIW). The previous characterisation utilised a truncated BRD9 protein, however, a chemoproteomic assay using cell lysate revealed comparable activity with the native protein (IC_50_ 80 nM). Cellular activity was confirmed using a nanoBRET assay with the BRD9 Brd (IC_50_ 0.158 μM), and cellular permeability further demonstrated in an artificial membrane assay.

**I-BRD9** was found to lead to the selective modulation of a range of genes. Kasumi-1 cells were treated with **I-BRD9** (10 μM) or the BET inhibitor **I-BET151** (ref. 85) (1 μM) to investigate the difference between BRD9 and BET inhibition. 700 genes showed up- or down-regulation (>1.5-fold expression change relative to DMSO vehicle) by **I-BRD9** treatment but not by **I-BET151**. Of these, the modulation of four genes implicated in cancer and immunological pathways (*CLEC1*, *DUSP6*, *FES* and *SAMSN1*) were subsequently confirmed by qPCR. This broad modulation of gene expression by inhibition of Brd binding confirms the epigenetic nature of the BRD9 protein and warrants further exploration.

A further dual BRD7/9 probe was developed by Hay *et al.* at the University of Oxford and the SGC. An indolizine active against the Brd of BAZ2B was also found to have activity against BRD9; following a biophysical assay-guided SAR program, compound **26** was identified (reference compound **28**, Fig. 12).^86^ This compound displayed a high affinity for BRD9 (*K*_*D*_ 68 nM) and BRD7 (*K*_*D*_ 0.368 μM), with no affinity for the original target BAZ2B. Selectivity amongst Brds was further assessed by a DSF assay, where only BRD9 (Δ*T*_m_ 4.5 °C) and BRD7 (Δ*T*_m_ 5.6 °C) demonstrated a thermal shift >2.0 °C after treatment with compound **26**. For BRD9, binding was found to be driven by enthalpic contributions (Δ*H* –10.7 kcal mol^–1^, *T*Δ*S* –1.32 kcal mol^–1^) whereas entropy dominated BRD7 binding (Δ*H* –2.64 kcal mol^–1^, *T*Δ*S* 5.99 kcal mol^–1^). An X-ray co-crystal structure of compound **26** with BRD9 revealed the methyl ketone formed the conserved H-bonds of KAc recognition to N216 and Y163, with additional π–π stacking interactions between the indolizine system and Y222 (PDB ID ; 5E9V). Final characterisation through a FRAP assay showed compound **26** decreased the recovery time of BRD9-GFP in a dose-dependent manner, towards the *t*_1/2_ observed with a non-binding N216F mutant, confirming cellular permeability and activity.

A pair of related BRD9 and BRD7/9 probes were developed by Martin and co-workers at Boehringer Ingelheim and the SGC. Parallel screening by biophysical assays and computational docking identified a dimethylpyridinone lead, which, after a structure-based drug discovery program, resulted in the discovery of the naphthyridinone-based probes **BI-7273** and **BI-9564** (Fig. 12).^87^ **BI-9564** showed selectivity for BRD9 (*K*_*D*_ 5.9 nM) over BRD7 (*K*_*D*_ 0.239 μM), whereas **BI-7273** showed a decreased selectivity profile (BRD9 IC_50_ 19 nM; BRD7 IC_50_ 0.117 μM). Wider selectivity screening by DSF with **BI-7273** showed significant binding to BRD7 (Δ*T*_m_ 9.7 °C), BRD9 (Δ*T*_m_ 11.4 °C) and CECR2 (Δ*T*_m_ 8.2 °C), an observation mirrored by **BI-9564** (BRD7 Δ*T*_m_ 6.5 °C; BRD9 Δ*T*_m_ 9.2 °C; CECR2 Δ*T*_m_ 5.6 °C). The off-target affinity for CECR2 was confirmed by ITC for both **BI-7273** (CECR2 *K*_*D*_ 0.187 μM) and **BI-9564** (CECR2 *K*_*D*_ 0.2 μM), although the latter affinity was over 30-times higher than that for BRD9. Aside from these three domains, both compounds showed low affinity for all other Brds assessed, including for the BET family of proteins. In wider target screening, some limited activity was observed with a number of kinases (IC_50_ 3.8–5.1 μM) and GPCRs (GPCRs IC_50_ ≥ 10 μM). Co-crystal structures of **BI-9564** (PDB ID ; 5F1H) and **BI-7273** (PDB ID ; 5EU1) with BRD9 were determined by X-ray crystallography, demonstrating the carbonyl of the naphthyridinone replicating the key H-bonds of KAc recognition to N216 and Y173, in addition to numerous π-interactions with the protein. Cellular target engagement was demonstrated through a FRAP assay, with complete inhibition of BRD7/9-chromatin binding by both probes at 1 μM. Importantly, no cellular activity against the off-target Brd CECR2 was observed at this concentration. Further evidence of cellular activity of **BI-7273** was demonstrated through disruption of BRD9-histone H3 interactions in a nanoBRET assay at submicromolar concentrations.^88^ A final cytotoxicity assay of the compounds showed no effect on proliferation after 24 hours of exposure.

**BI-7273** and **BI-9564** were found to have activity against acute myeloid leukemia (AML) cell lines. Whilst screening the probes against a range of cancer cells lines, **BI-9564** was found to induce growth inhibition in a number of AML cell lines, with exposure to **BI-7273** found to result in partial but significant inhibition of *Myc* expression in these cells.^87^ The role of BRD9 in supporting leukemia-maintenance through the SWI/SNF complex and *Myc* expression has been subsequently confirmed through genetic knockdown studies.^88^ To confirm the role of Brd binding in this antiproliferative activity, domain-swapped alleles expressing BRD9 with the Brd of another protein were developed. It was fortuitiously discovered that swapping the Brd with BRD4(1) led to a retention of the chromatin binding specificity and activity of the native protein. AML cells expressing these domain-swapped proteins showed full BRD9 function, despite the different Brd architecture. Treatment of these cells with **BI-7273** showed a complete resistance to the antiproliferative effects of the probe, confirming both the on-target selectivity of **BI-7273** and the identification of BRD9 as the sole mediator of this anti-proliferative activity. In the same experiment, this allele only partially reduced the anti-proliferative activity of **I-BRD9**, and had a minimal effect on sensitivity to **LP99**, suggesting some off-target effects of both these probes. These data show the power of domain-swap experiments as a general strategy to demonstrate on- and off-target activity of probes in cells, and should be incorporated into the routine characterisation of Brd probes where possible. Human acute myeloid eosinophilic leukemia cell line EOL-1 proved to be the most susceptible to inhibition with **BI-7273** (EC_50_ 0.8 μM) and **BI-9564** (EC_50_ 1.4 μM), with BRD9 confirmed as the biological target through a domain-swap experiment that mitigated all antiproliferative effects of **BI-9564** up to concentrations of 5 μM.^87^

Towards application in *in vivo* studies, the pharmacokinetic parameters of **BI-7273** and **BI-9564** were assessed. Good solubility, moderate to low hepatic clearance across different model systems, low plasma protein binding and no cytochrome P450 inhibition at concentrations below 50 μM were observed for both compounds, although significant efflux ratios in a Caco-2 transporter assay were observed. Initial testing in mouse models with twice daily *p.o.* dosing, at both 20 mg kg^–1^ and 180 mg kg^–1^, resulted in dose-dependent blood levels in excess of the EC_50_ determined for antiproliferative activity against EOL-1 cells. Of the two probes, **BI-9564** achieved both a higher exposure and bioavailability than **BI-7273**. A seven day tolerability study was performed on CIEA-NOG mice with daily *p.o.* dosing of **BI-9564** at a 180 mg kg^–1^ loading, which showed good tolerance and minimum weight change observed.

The efficacy of **BI-9564** as a treatment for AML was explored further in a disseminated mouse model. EOL-1 cells, transduced with a luciferase-expressing vector to allow for a bioluminescent assessment of tumour load, were injected into CIEA-NOG mice, and **BI-9564** was subsequently administered *p.o.* daily. Plasma samples revealed a high systemic exposure of **BI-9564**, with mean total plasma concentrations in excess of the EC_50_ required for EOL-1 cellular proliferation inhibition, for 20 h after dosing. A statistically significant reduction in tumour growth, measured in average radiance, was observed, resulting in median tumour growth inhibition of 52%. This was confirmed by imaging data in which the disease burden was visually reduced. In addition to decreased tumour growth, there was a small increase in median survival of the treatment group compared to the vehicle animals. Although only moderate effects on AML proliferation were observed, these experiments demonstrated the suitability of this probe for further *in vivo* assessment of BRD7 and BRD9 inhibition.

Recently a novel BRD7/9 probe has been added to the SGC chemical probes list with limited data. **TP-472**, which features a pyrrolo[1,2-*a*]pyrimidine scaffold and a methyl ketone as a KAc bioisostere, was developed collaboratively between Takeda and the SGC (Fig. 12).^89^ **TP-472** has reportedly demonstrated a high potency for BRD9 (*K*_*D*_ 33 nM) and BRD7 (*K*_*D*_ 0.34 μM), with >30-fold selectivity over other Brds. Cellular activity has been demonstrated through a nanoBRET assay with BRD9 (EC_50_ 0.32 μM). A structurally-related negative control compound (BRD9 *K*_*D*_ > 20 μM) has also been developed for use in parallel in biological assays to corroborate on-target activity. A full report of the development and characterisation of **TP-472** is awaited in the scientific literature.

Other potent ligands of BRD7 and BRD9 have been reported, however these lack the potency, selectivity and/or cellular activity requisite for use as chemical probes. From fragment screening, the purine **27** (reference compound **11**, Fig. 12) was developed from a structure-based drug discovery program.^90^ This ligand displayed a reasonable affinity for BRD9 (*K*_*D*_ 0.278 μM), and good selectivity over the representative BET protein BRD4(1) (*K*_*D*_ 1.4 μM). Cellular activity of the ligand was confirmed through a nanoBRET assay (IC_50_ 0.477 μM), and no cytotoxicity in HEK293 cells were seen up to concentrations of 33 μM. Conversely, a platform-based approach screening derivatives of the known KAc bioisostere [1,2,4]triazolo[4,3-*a*]phthalazine resulted in the identification of compound **28** (reference compound **51**, Fig. 12).^91^ Moderate affinities were observed equally for BRD9 (IC_50_ 0.199 μM), CBP (IC_50_ 0.199 μM) and BRD4(1) (IC_50_ 0.158 μM). Inhibition of the interaction of a CBP Brd-GFP fusion protein with chromatin was demonstrated with a FRAP assay, confirming cellular permeability and activity of compound **28**.

### BRPF1/2/3

**Fig. 13:**
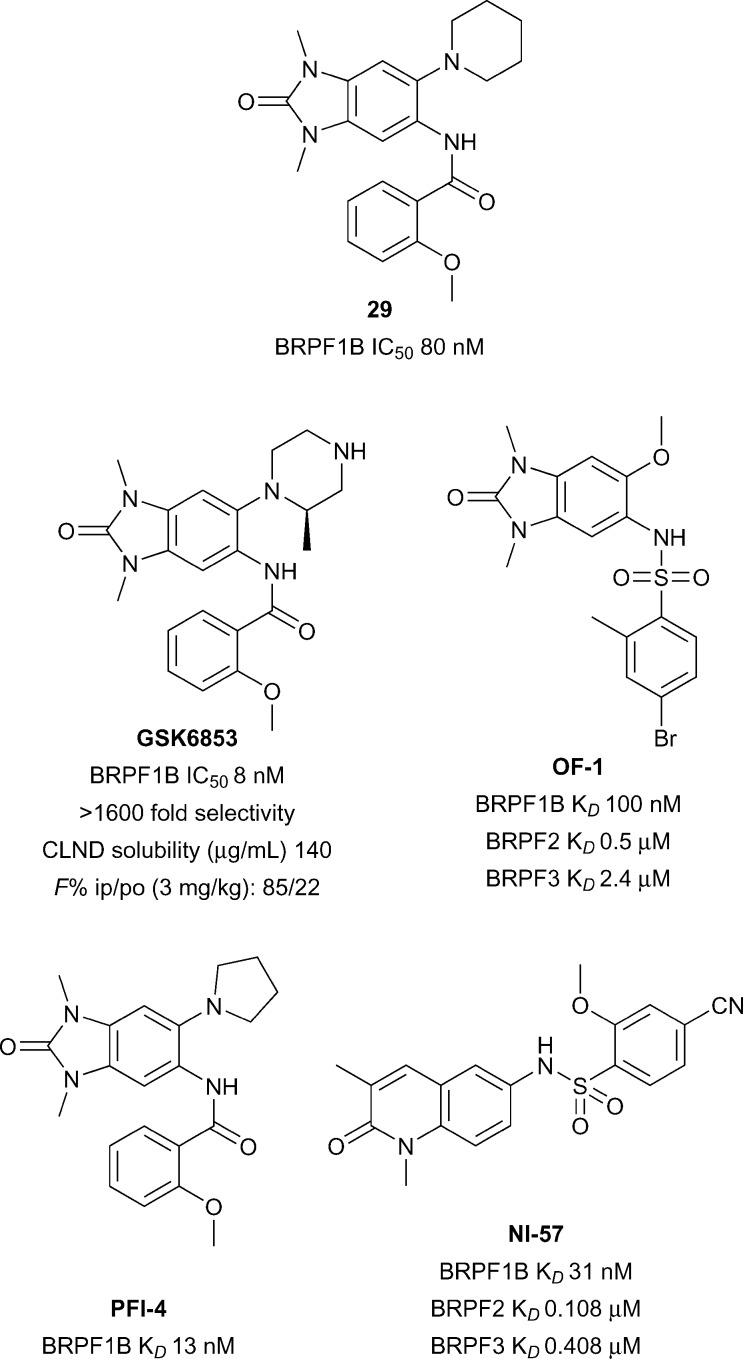
BRPF1B inhibitors.

The bromodomain-PHD finger protein (BRPF) aids the complex assembly of MYST-family histone acetyltransferases (HATs).^92^ BRPF1 forms a subunit of the monocytic leukemic zinc finger (MOZ) complex in which translocations have been linked to aggressive forms of myeloid leukemia.^93^ Relatively little is known about the biological function or therapeutic potential of the Brd of BRPF1, however BRPF1B has been predicted to be a highly druggable target.^28,94^ GSK recently reported the discovery of BRPF1B inhibitor *N*,*N*-dimethylbenzimidazolone compound **29** (reference compound **34**, Fig. 13).^95^

Compound **29** was further optimized to give the a potent and selective BRPF1 Brd inhibitor **GSK6853** with improved solubility.^96^ **GSK6853** (BRPF1B IC_50_ 8 nM) showed 1600-fold selectivity against other bromodomains in the DiscoveRx BROMO*scan* panel. Furthermore, **GSK6853** showed excellent cellular activity in a BRPF1B NanoBRET assay against NanoLuc tagged full-length BRPF1 bromodomain (IC_50_ 20 nM) and a favourable solubility profile (140 μg mL^–1^) supporting its potential utility for *in vivo* work. Earlier reports of BRPF inhibitors also include the structurally related **PFI-4** and **OF-1**.^97,98^ Another BRPF chemical probe **NI-57** features an orthogonal 1,3-dimethylquinolin-2(1*H*)-one chemotype.^99^

### ATAD2

**Fig. 14:**
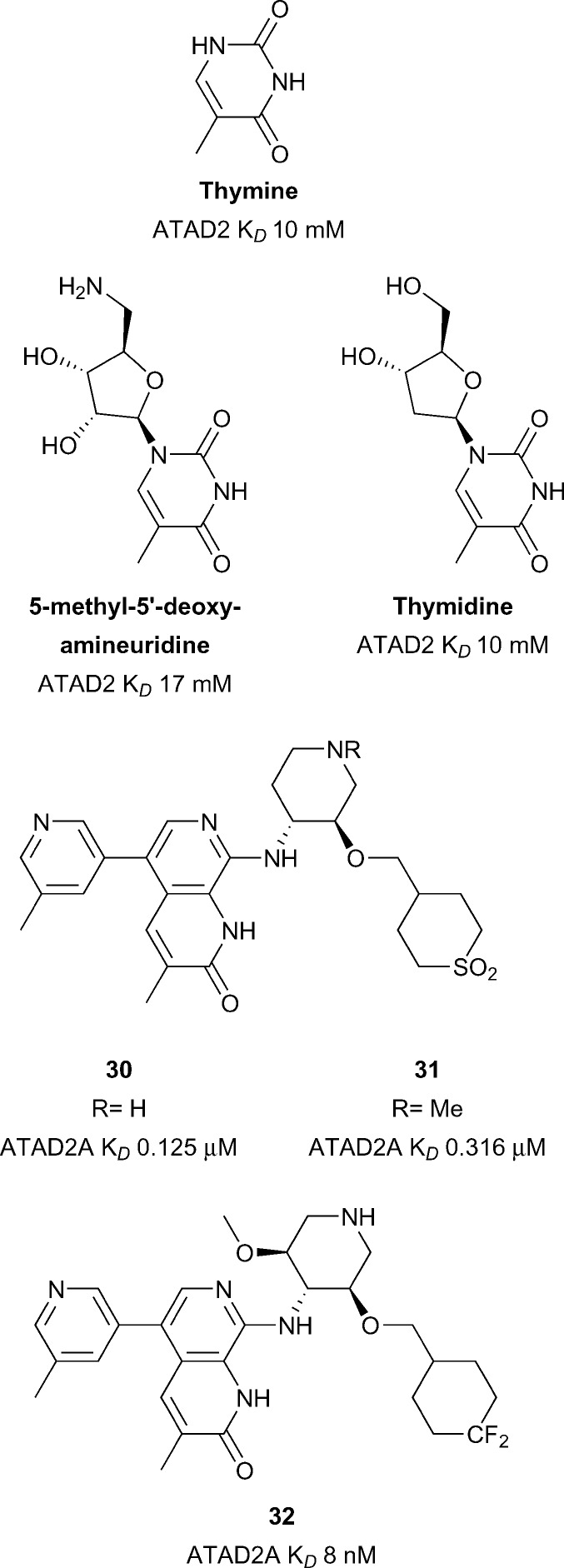
ATAD2 inhibitors.

Overexpression of the Brd-containing protein ATAD2 (ATPase family, AAA domain containing 2) has been linked to a variety of cancers including breast,^100,101^ prostate,^102^ liver,^103^ lung,^100^ osteosarcoma^104^ among others.^105^ Little is known of the role that the ATAD2 bromodomain plays in these indications. ATAD2(A/B) have been predicted to be difficult in terms of druggability analysis.^28^ Early work towards the development of inhibitors of ATAD2 focused on the use of crystal transfer/soaking approaches in parallel to NMR screening of nucleoside derived fragments (Fig. 14).^106^ Chaikuad *et al.* obtained nine crystal structures of ATAD2 in complex with fragments such as **thymidine** (ATAD2 *K*_*D*_ 10 mM). These nucleoside analogues represent chemical starting points for the development of more potent ATAD2 inhibitors. More recent work from Demont *et al.* describes the development of the first known micromolar inhibitors of the ATAD2 bromodomain through a focused fragment based screen.^105^ Optimisation of this fragment series led to compounds **30** and **31** (reference compounds **38** and **46**, Fig. 14) displaying very good potency for the ATAD2 bromodomain (**30** ATAD2 *K*_*D*_ 0.125 μM, **31** ATAD2 *K*_*D*_ 0.316 μM) (Fig. 14).^107^ Compounds **30** and **31** showed good selectivity against the therapeutically implicated BET bromodomains however cell permability needs to be improved. Compounds **30** and **31** were used as lead compounds in the development of a cell-permeable, potent and chemical probe of the ATAD2 bromodomain resulting in compound **32** (reference compound **16**) reported by the same group from GSK.^108^ Compound **32** displays good potency (ATAD2 *K*_*D*_ 8 nM), selectivity (2.8 log selectivity against BET Brds) and moderate cellular activity (IC_50_ 2.7 μM) in a NanoBRET assay against NanoLuc tagged truncated ATAD2 bromodomain. Compound **32** was shown to target both ATAD2A and ATAD2B using DiscoveRx's *BROMOScan* (ATAD2A *K*_*D*_ 1.3 nM, ATAD2B *K*_*D*_ 1 nM). The enantiomer of compound **32** displays much weaker activity against ATAD2 (ATAD2 *K*_*D*_ 3.1 μM) and so is a potential negative control, also displayed in a much weaker inhibition profile in the same NanoBRET assay. Novel use of a –CF_2_ group as a polar hydrophobic isostere of the sulfone groups seen in compounds **30** and **31** allowed for improvements in both selectivity, solubility and cell permeability. It is noteworthy that although discovered independently, the ATAD2 Brd inhibitors in Fig. 14 share a common unsaturated, 3-methyl substituted lactam KAc mimetic motif.

## Sub-family V

### BAZ2A/B

The BAZ proteins (Bromodomain Adjacent to Zinc finger) represents a diverse set of proteins including BAZ1A, BAZ1B, BAZ2A and BAZ2B.^109^ Both BAZ2A and BAZ2B have been shown to be involved in chromatin remodelling^110^ and regulation of non-coding RNAs.^111^ Mutations in the BAZ2B gene has been linked to sudden cardiac death^112^ and over expression of BAZ2B negatively affects the outcome of pediatric B cell acute lymphoblastic leukemia (B-ALL).^113^

**Fig. 15:**
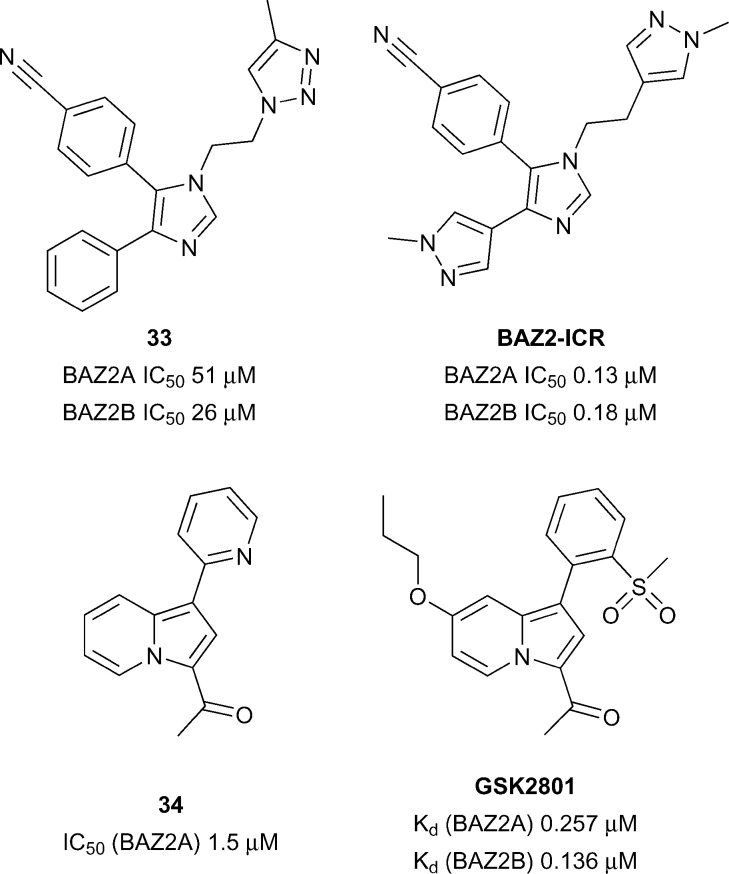
BAZ2A/BAZ2B inhibitors.

Aberrant overexpression of BAZ2A correlates well with recurrence in prostate cancer and is also linked with maintaining prostate cancer cell growth.^114^ BAZ2B has been predicted to be difficult in terms of druggability analysis.^28^ Owing to the significant therapeutic potential in developing BAZ2A/BAZ2B inhibitors/chemical Drouin *et al.* ^113^ sought to develop a potent, selective and cell active chemical probe through structure based discovery starting from hit compound **33** (reference compound **1**, Fig. 15). Through an iterative process of structure based design BAZ2A/BAZ2B inhibitor, **BAZ2-ICR** was discovered displaying potency (BAZ2A IC_50_ 0.13 μM, BAZ2B IC_50_ 0.18 μM) and good selectivity against a wide panel of Brds.

Furthermore, **BAZ2-ICR** showed accelerated recovery in a FRAP assay at 1 μM for GFP-tagged full length BAZ2A comparable to a non-histone binding mutant negative control. A chemically distinct BAZ2A/BAZ2B inhibitor was disclosed in the form of compound **GSK2801** (Fig. 15). **GSK2801** was discovered through structure based design starting from initial hit compound **34** (reference compound **1**, Fig. 15) and displays high potency for BAZ2A/BAZ2B (BAZ2A IC_50_ 0.257 μM, BAZ2B IC_50_ 0.136 μM), good selectivity against other Brds and target engagement in a cellular FRAP assay using full-length GFP tagged BAZ2A.^115^ In addition, **GSK2801** displayed favourable pharmacokinetic properties after intraperitoneal and oral dosing to male CD1 mice providing rationale for **GSK2801** to be used as an *in vivo* as well as *in vitro* BAZ2A/BAZ2B inhibitor.

To date, no inhibitors have been reported for the remaining BAZ proteins BAZ1A and BAZ1B. Although BAZ1A and BAZ1B have a similar domain architecture, their Brds are actually very different from BAZ2A/2B and each other.

### TRIM24

**Fig. 16:**
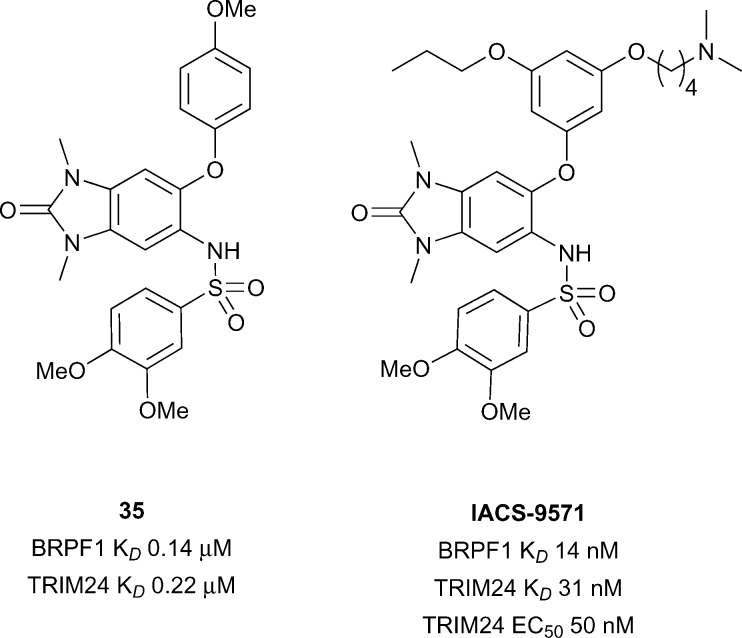
Dual TRIM24/BRPF1 inhibitors.

A family of bromodomain containing proteins, the tripartite motif containing proteins (TRIMs), more specifically TRIM24, TRIM28 and TRIM33, represent interesting targets due to their role in a variety of cancers including breast,^116,117^ head and neck,^118^ non-small-cell lung,^119^ hepatocellular,^120^ and glioblastoma.^121^ TRIM24 has been predicted to be difficult in terms of druggability analysis*.* ^28^ Recent work from the SGC and Bayer led to the development of a dual BRPF1B/TRIM24 Brd inhibitor.^122^ Screening of commercial 1,3-benzimidazolones led to the discovery of compound **35** (reference compound **34**, Fig. 16) showing good binding affinity for BRPF1B and TRIM24 Brds (BRPF1B *K*_*D*_ 0.14 μM, TRIM24 *K*_*D*_ 0.22 μM). Compound **35** displayed cellular target engagement for TRIM24 *via* a FRAP assay at 1 μM concentration for full length TRIM24 fused to GFP. Another report by Palmer *et al.* ^123^ showed optimisation of the same 1,3-benzimidazolone motif of compound **35** leading to the discovery of **IACS-9571**. **IACS-9571** displayed excellent potency against both BRPF1B and TRIM24 (BRPF1B *K*_*D*_ 14 nM, TRIM24 *K*_*D*_ 31 nM), highly potent cellular activity for TRIM24 (TRIM24 EC_50_ 50 nM – Cellular AlphaLisa using Flag-tagged TRIM24-PHD-Bromo construct) and good pharmacokinetic properties (*F* 29% after oral dosing). Despite progress in the area, a selective TRIM24 Brd inhibitor has not yet been reported.

## Sub-family VI

The 6th sub-family of Brds is composed of only two members, MLL and TRIM28. MLL is a large protein with many functional domains including a Brd and is one of the most frequently mutated genes in cancer.^124^ TRIM28 has been associated with regulation of mitophagy^125^ and HCMV latency.^126^ Neither of these Brds has any reported inhibitors despite their interesting links to disease, presumably due to the challenge in finding hit compounds for their atypical KAc binding residues (Asp in MLL and Thr in TRIM28).

## Sub-family VII

**Fig. 17:**
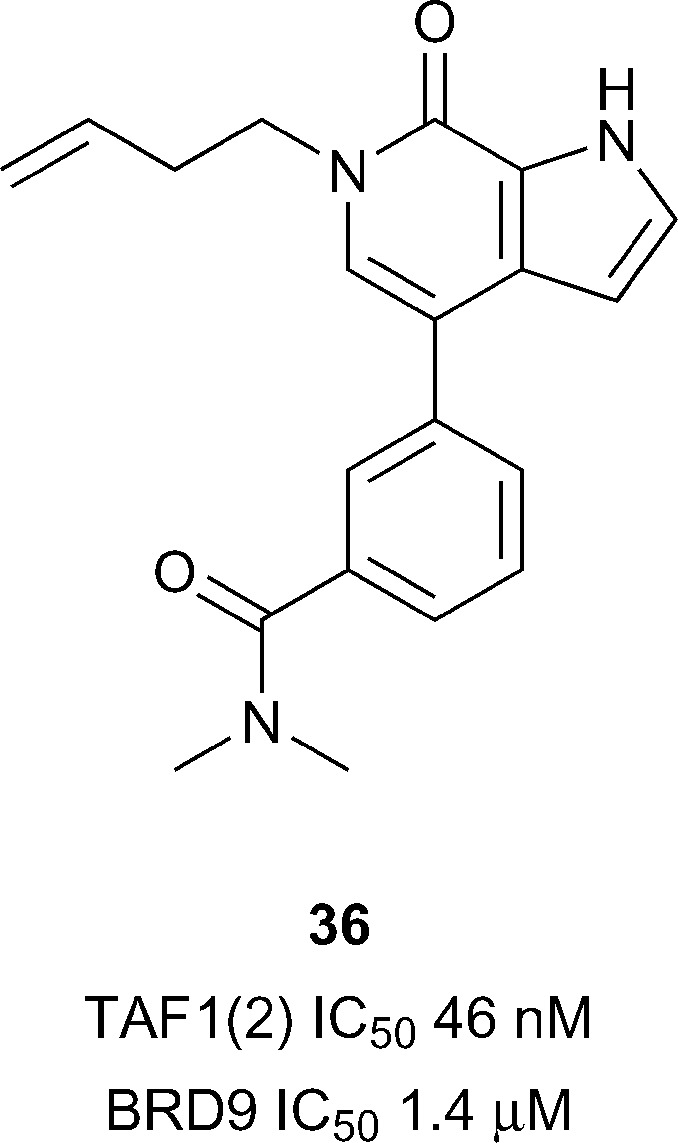
TAF1(2) inhibitor compound **40** sub-family VIII.

TAF1 has been predicted to be difficult in terms of druggability analysis*.* ^28^ From the previously mentioned report by Genentech and Constellation pharmaceuticals^33^ a highly potent TAF1(2) inhibitor, compound **36** (reference compound **5**, Fig. 17), was accessed through the development of *N*-methyl pyrrolopyridones. Compound **36** was shown to inhibit TAF1(2) Brd with excellent potency (IC_50_ 46 nM), 30-fold selectivity over BRD9 (IC_50_ 1.4 μM) and displayed novel interactions stemming from the rearrangement of the conserved solvent network. The 1-butenyl substituent extends into the water channel between Tyr1540 and lipophilic shelf residues Pro1527 and Phe1528 displacing conserved water 4 in the KAc binding site. Compound **36** represents an attractive lead for development of additional TAF1 and TAF1L inhibitors.

### SMARCA/PB1

The final bromodomain sub-family is composed of PB1 (Polybromodomain protein 1), SMARCA2 (SWI/SNF related, matrix associated, actin dependent regulator of chromatin, subfamily A 2; BRM) and SMARCA4 (BRG1). These three Brd-containing proteins are components of human SWI/SNF (switch/sucrose nonfermentable) chromatin remodelling complexes and have been linked genetically to a number of cancers.^127,128^

**Fig. 18:**
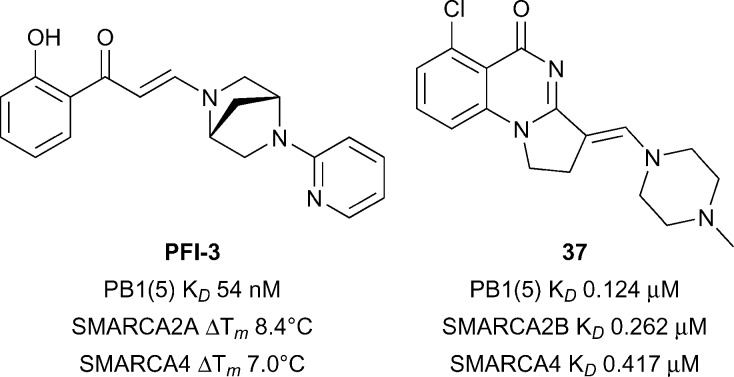
PB1/SMARCA2/SMARCA4 inhibitors.

PB1 is a multidomain protein containing six different Brds whereas SMARCA2 and SMARCA4 each have a single Brd and an ATPase domain. In order to dissect the role of the bromodomain contribution to SWI/SNF mediated processes, chemical inhibitors targeting the bromodomains of SMARCA2/SMARCA4/PB1 represent useful entities. PB1(A/B/C/5) has been predicted to be intermediate in terms of druggability analysis, SMARCA4/28 have been predicted to be difficult.^28^ Gerstenberger *et al.* reported the discovery of **PFI-3** (Fig. 18), a broadly selective, potent and cellular active inhibitor of family VIII bromodomains including SMARCA2/SMARCA4/PB1.^129^ **PFI-3** showed excellent binding affinities for PB1 (PB1(5) *K*_*D*_ 54 nM), and SMARCA2/SMARCA4 (*K*_*D*_ < 0.1 μM) and excellent selectivity for these sub-family VIII bromodomains in a panel against >40 bromodomains from other families.^130–132^ **PFI-3** also displayed an increase in the half recovery time using a FRAP assay (1 μM **PFI-3**) comparing recovery times of wild-type full-length SMARCA2 *vs.* a mutant incapable of binding chromatin (N1464F) providing evidence for cellular target engagement. No inhibitory effects were seen against a range of cellular endpoints in 12 primary human cell based systems when incubated with **PFI-3**.^130^ More recently Sutherell *et al.* at the University of Cambridge and the SGC reported the structure guided discovery of compound **37** (reference compound **26**, Fig. 18) which showed good potency for PB1(5) (*K*_*D*_ 0.126 μM), SMARCA2B (*K*_*D*_ 0.262 μM), and SMARCA4 (*K*_*D*_ 0.417 μM).^133^ Compound **42** showed reasonable selectivity over other bromodomain families, cellular target engagement at 1 μM with full-length SMARCA2 (FRAP assay) and revealed a new interaction in a crystal structure with PB1(5) through potential halogen bonding with Met731. Used in concert with **PFI-3** these inhibitors may be used to decipher the role of sub-family VIII Brds.

## Conclusions

Since the initial disclosure of the first BET inhibitors, Brds have been a target class of great interest in drug discovery. The success in discovering small molecule inhibitors targeting half of the Brd family in less than a decade has greatly expanded our understanding of the biological role of the individual Brds and jump-started translational efforts, primarily in oncology.

Although half of the Brds have succumbed to inhibitor discovery efforts, another half remains. For many of these, potent inhibitors may already exist and broader cross-screening may uncover additional Brd activity *e.g.* ATAD2A inhibitor **32** also inhibits ATAD2B; and the PCAF inhibitor **4** may also inhibit the closely related GCN5. For other typical Brds (BRD8, WRD(1), PHIP(1), BRWD3(1)) cross-screening of focused Brd inhibitor sets may deliver hits. But there remain a further twelve atypical Brds (Fig. 1B and C) for which no hits exist. New unbiased screening efforts using fragments,^67^ HTS,^134^ DNA-encoded libraries,^135^ or *in silico* screening,^63^ will be needed to find new chemotypes targeting Tyr, Thr and Asp-containing Brds.
